# Humanized Mouse Model of Skin Inflammation Is Characterized by Disturbed Keratinocyte Differentiation and Influx of IL-17A Producing T Cells

**DOI:** 10.1371/journal.pone.0045509

**Published:** 2012-10-19

**Authors:** Vivian L. de Oliveira, Romy R. M. C. Keijsers, Peter C. M. van de Kerkhof, Marieke M. B. Seyger, Esther Fasse, Lars Svensson, Markus Latta, Hanne Norsgaard, Tord Labuda, Pieter Hupkens, Piet E. J. van Erp, Irma Joosten, Hans J. P. M. Koenen

**Affiliations:** 1 Laboratory of Medical Immunology, Department of Laboratory Medicine, Radboud University Nijmegen Medical Centre, Nijmegen, The Netherlands; 2 Dermatology Department, Radboud University Nijmegen Medical Centre, Nijmegen, The Netherlands; 3 Department of Plastic Surgery Radboud University Nijmegen Medical Centre, Nijmegen, The Netherlands; 4 Department of Disease Pharmacology, LEO Pharma, Ballerup, Denmark; 5 Department of Molecular Biomedicine, LEO Pharma, Ballerup, Denmark; Statens Serum Institute, Denmark

## Abstract

Humanized mouse models offer a challenging possibility to study human cell function *in vivo*. In the huPBL-SCID-huSkin allograft model human skin is transplanted onto immunodeficient mice and allowed to heal. Thereafter allogeneic human peripheral blood mononuclear cells are infused intra peritoneally to induce T cell mediated inflammation and microvessel destruction of the human skin. This model has great potential for *in vivo* study of human immune cells in (skin) inflammatory processes and for preclinical screening of systemically administered immunomodulating agents. Here we studied the inflammatory skin response of human keratinocytes and human T cells and the concomitant systemic human T cell response.

As new findings in the inflamed human skin of the huPBL-SCID-huSkin model we here identified: 1. Parameters of dermal pathology that enable precise quantification of the local skin inflammatory response exemplified by acanthosis, increased expression of human β-defensin-2, Elafin, K16, Ki67 and reduced expression of K10 by microscopy and immunohistochemistry. 2. Induction of human cytokines and chemokines using quantitative real-time PCR. 3. Influx of inflammation associated IL-17A-producing human CD4+ and CD8+ T cells as well as immunoregulatory CD4+Foxp3+ cells using immunohistochemistry and -fluorescence, suggesting that active immune regulation is taking place locally in the inflamed skin. 4. Systemic responses that revealed activated and proliferating human CD4+ and CD8+ T cells that acquired homing marker expression of CD62L and CLA. Finally, we demonstrated the value of the newly identified parameters by showing significant changes upon systemic treatment with the T cell inhibitory agents cyclosporine-A and rapamycin.

In summary, here we equipped the huPBL-SCID-huSkin humanized mouse model with relevant tools not only to quantify the inflammatory dermal response, but also to monitor the peripheral immune status. This combined approach will gain our understanding of the dermal immunopathology in humans and benefit the development of novel therapeutics for controlling inflammatory skin diseases.

## Introduction

The *in vivo* study of human biological processes is severely limited by ethical and technical constraints. An attractive relevant alternative is the use of humanized mice or “mouse-human chimaeras”. Humanized mice are immunodeficient mice that are engrafted with human tissue or cells, such as hematopoietic stem cells (HSCs) or peripheral blood mononuclear cells (PBMC). Humanized mice offer a valuable tool in pre-clinical drug testing in translational medicine and investigations of human biology in areas like cancer, (auto-) inflammation/immunity, infectious diseases and immunotherapy [1], [2].

Several humanized mouse models have been described to study T cell mediated skin diseases [3]–[7]. In these models, healthy human skin, diseased-prone skin or bioengineered skin is transplanted onto immunodeficient mice, allowed to become vascularized and heal, and in some of these models human immune cells are infused that will reconstitute the recipient with human immune cells to induce skin inflammation [5], [6]. Although all these models have advantages and disadvantages their application to drug discovery and for the development of cellular therapies has already proven to be fruitful [8]–[11].

To study the local inflammatory as well as the systemic human T cell response *in vivo* we focused on the human peripheral blood lymphocyte reconstituted severe combined immunodeficient mouse (SCID) human skin allograft model ( huPBL-SCID-huSkin model ) initially described by Pober's group [5], [6]. In this model human skin is transplanted on to immunodeficient SCID/beige mice, and since these recipients lack functional mature T and B cells and have impaired NK cell- and macrophage function [12], [13], human skin is revascularized and accepted. After healing of the human skin, allogeneic human PBMC are infused intra peritoneally (ip), resulting within 2–3 weeks in microvascular cell injury and human T cell infiltration of the human skin [5], [6]. This model is of particular interest to study the local pathology of skin inflammation. For this purpose it is crucial to be able to quantify the cutaneous inflammatory response by clinical relevant parameters associated with dermal inflammation, such as inflammation-associated deregulated expression of keratinocyte differentiation markers and characterization and enumeration of skin infiltrating human lymphocytes. This information has not previously been published regarding this model.

Most studies on inflammation in humanized mouse models focus on the local site of inflammation. A caveat in these humanized mouse models is the study of the systemic immunological response, which besides the local site of inflammation is crucial in the generation and regulation of the immune program (e.g, effector Thelper Th1, Th2, Th17, or regulating suppressor Treg). Important steps in mounting a T cell immune response include T cell activation, differentiation and expansion in the draining lymph nodes. Activated T cells leave the lymph node via the efferent lymphatics and enter the circulation through the lymph-vascular system and depending on their homing imprint the cells migrate into tissues or colonize other immune compartments [14], [15]. Moreover, inflammatory skin diseases, such as moderate to severe psoriasis, are traditionally treated with systemic medication such as methotrexate, retinoids, and cyclosporine A and more recently with biologicals such as anti-TNF or anti-p40 therapy. This systemic treatment will not only influence the local skin immune response, but will also interfere systemically with T cell activation, differentiation, expansion and homing. At present, little Information is available on the effects of systemic medication of immune modulating agents and their effects on the systemic immune response in humanized mouse models. This information is crucial to identify treatment-related systemic biomarkers for immunomonitoring of patients undergoing clinical trials.

In the present study, both local human skin inflammatory processes as well as systemic human CD4+ and CD8+ T cell responses in the huPBL-SCID-huSkin model were studied. As new findings in this model we identified relevant markers of human dermal pathology such as aberrant expression of hBD2, Elafin, K10 and K16, enabling quantification of the local skin inflammatory response by keratinocytes, markers for T cell mediated responses in the skin, as well as, chemokine and cytokine induction and analysis of IL-17A-producing and Foxp3+ T cells. Also, we determined markers that enable quantitative analysis of systemic immune activation responses. Human skin inflammation was paralleled by the presence of CD4+Foxp3+ T cells, suggesting that immune regulatory pathways are serving to limit human tissue inflammation. Unraveling these pathways of human cells in this *in vivo* model will undoubtedly offer novel therapeutic strategies for controlling autoimmune tissue responses.

## Results

### Acanthosis and aberrant epidermal marker expression of hBD2, Elafin, K10 and K16 in the inflamed human skin in the huPBL-SCID-huSkin allograft model

Here we set out to define and quantify clinical relevant dermal parameters in the inflamed human skin the huPBL-SCID-huSkin allograft model as initially described by Pober's group [5], [6]. The model in brief, after grafting and healing of human skin onto immunodeficient SCID beige recipients, allogeneic human PBMC are infused intra peritoneally (i.p.), which results in microvessel destruction and human T cell influx of the human skin graft [5], [6].

**Figure 1:**
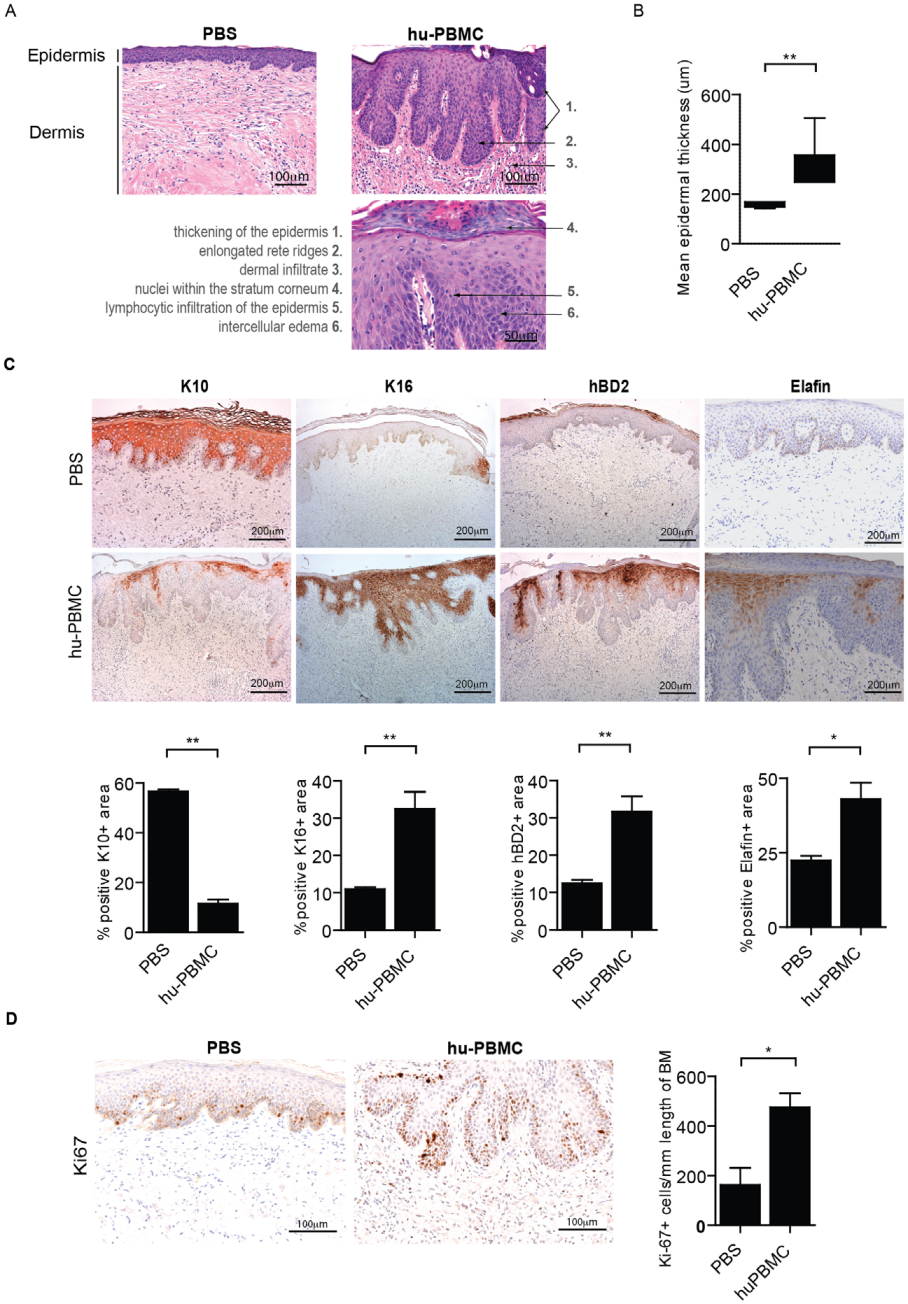
Acanthosis and aberrant epidermal marker expression of hBD2, Elafin, K10 and K16 in the inflamed human skin of the huPBL-SCID-hu Skin allograft model. **A.** Histology (H&E staining) of human skin grafts from SCID beige mice 21 days after infusion (i.p.) of huPBMC. Representative photographs are shown after PBS (left photograph) and huPBMC infusion (right photograph). Photograph shows increased epidermal thickness (acanthosis) and elongated fingerlike epidermal projections into the dermis (rete ridges) and large lymphocyte infiltration after huPBMC infusion. Abnormal presence of nuclei in the stratum corneum (parakeratosis) and infiltration of lymphocytes in the epidermis (exocytosis) (bottom-right photograph). 20×, 20×, 40× magnifications respectively. **B.** Quantitative microscopic histological analysis of the epidermal thickness (µm) of human skin grafts following infusion of PBS and huPBMC. Mean±SEM are shown for n = 3 and 6 upon PBS and huPBMC infusion resp. **C.** Immunohistochemistry of K10, K16, hBD2 and Elafin (brown) in human skin grafts from SCID beige mice 21 days after infusion (i.p.) of PBS (top panels) or huPBMC (lower panels). Photograpsh show representative examples, summarized data are given in the figures. Mean±SEM percentages of the area positive for the indicated markers is shown for n = 3 and 5–8 upon PBS and huPBMC infusion resp. 10× magnification. **D.** Ki-67 expression (brown) in the stratum basale of the epidermis. The insert shows a higher magnification. A representative example of n = 12 is shown. 20× magnification. Graphs show summarized data of Ki67+ cells/mm length of basement membrane after PBS or huPBMC infusion in human skin biopsies (mean±SEM, of n = 4 and 6, resp.).

Using histological microscopic examination of the human skin (**Fig. 1A**) we demonstrated hyperkeratosis (thickened keratinized upper layer), parakeratosis (nucleated keratinocytes in the cornified layer), acantosis (abnormal epidermal thickening, 155,1±10,6 vs 315,9±39,4 µm; PBS vs huPBMC, resp, p<0,01)(**Fig. 1B**), exocytosis (lymphocytes in the epidermis), spongiosys (intercellular edema between the keratinocytes and elongated rete ridges (fingerlike epidermal projections into the dermis) such as often observed in psoriatic lesions (**Fig. 1A**).

Using immunohistochemistry we quantitatively demonstrated that the changes in skin morphology were paralleled by a significant induction of keratinocyte associated inflammatory markers such as human β-defensin-2 (hBD2; 12,5±2,1 vs 31,7±10,9, p<0,01), Elafin (22,4±2,5 vs 43,0±14,7, p<0,05) and the hyper-proliferative marker keratin-16 (K16; 11,3±1,0 vs 38,6±12,5, p<0,01) , and down regulation of normal supra-basal keratin-10 (K10; 59,3±1,6 vs 11,6±4,9, p<0,01) (**Fig. 1C**).

Up-regulation of Ki67 expression by the keratinocytes indicated the presence of an increased number of dividing cells in the epidermis, which supports ongoing epidermal hyper-proliferation (**Fig. 1D**). Normal supra-basal K10 expression was found in the human skin transplants in the absence of PBMC infusion, indicating that the transplant procedure did not disturb normal supra-basal K10 expression (**Fig. 1C** **)**.

In summary, as a new finding we defined deregulated epidermal marker expression of hBD2, Elafin, K10, K16 and Ki67 in the inflamed human skin of the huPBL-SCID-huSkin model that can be employed to analyze the local inflammatory skin response in quantitative way.

### Human T cell infiltration and detection of human chemokines and cytokines in the inflamed human skin

**Figure 2:**
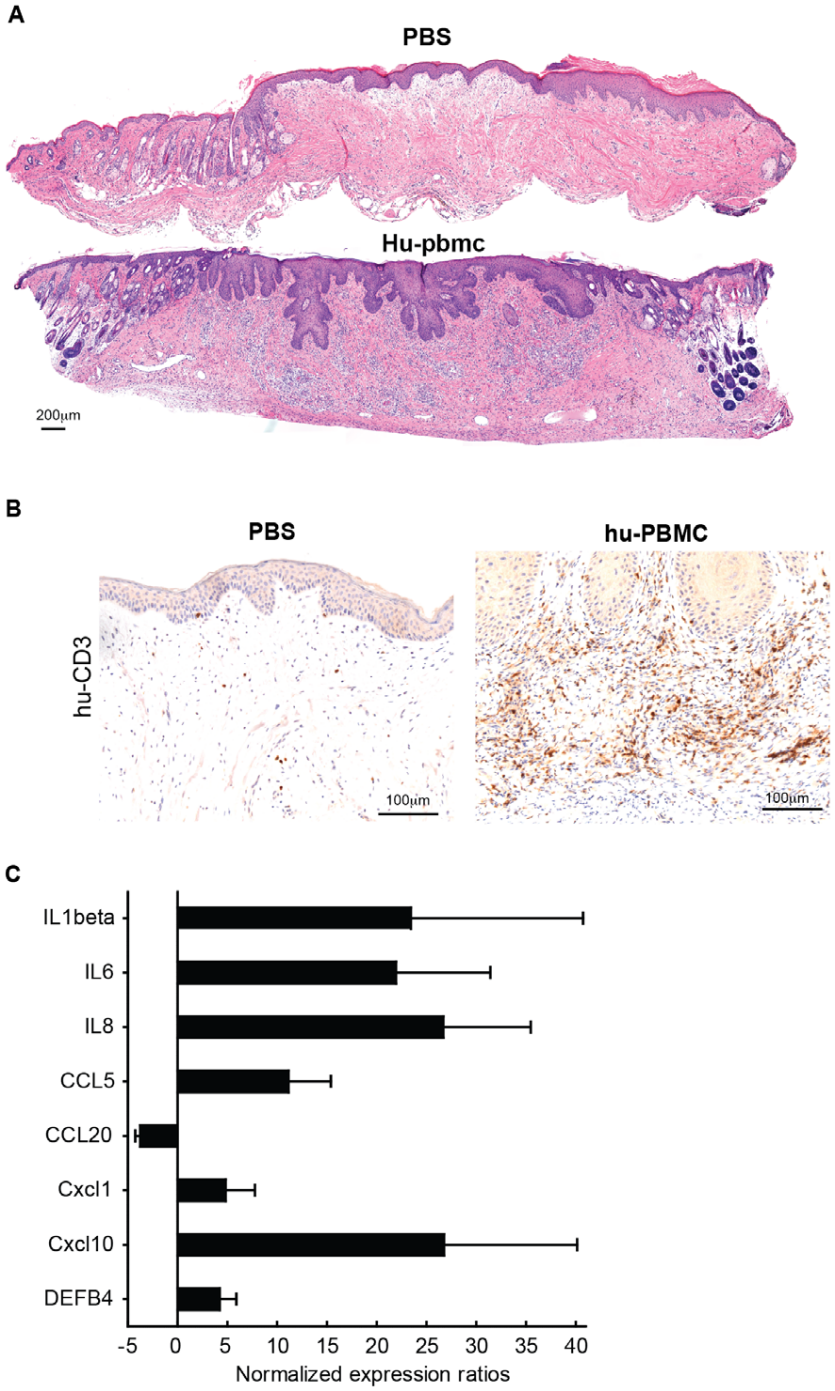
Induction of human chemokines and cytokines and influx of human T cells in the dermis and in the epidermis of huPBL-SCID-hu Skin allograft model. **A.** Images show representative panoramic overviews (H&E staining) of human skin grafts from SCID beige mice 21 days after infusion (i.p.) of PBS (top) or huPBMC (below). Note that human epidermis is thicker than the mouse epidermis (present in the left edge of the specimen in the top image and in both edges in the bottom image) and in contrast with human skin, mouse skin has closely spaced hair folicules troughout the epidermis. Images were composed using PTGui software (New House Internet Services B.V.; http://www.ptgui.com/). **B.** Immunohistochemistry of human CD3+ (brown) T cell infiltration in the human dermis and epidermis, 21 days after infusion of huPBMC. Representative example of n = 12 are shown. 20× magnification. **C.** Gene expression analysis using quantitative real-timePCR of human cytokine and chemokine transcripts in the human skin 21 days after infusion of huPBMC. Figure shows fold increase in cytokine and chemokine mRNA expression levels after huPBMC infusion as compared to PBS infusion (n = 5 and 3; huPBMC and PBS resp.).

Next to the epidermal changes described above, we found mononuclear cell infiltrates, including human CD3+ T cells, with a diffuse distribution throughout the human skin, but not in the mouse skin (**Fig. 2A+B**). Control mice that were transplanted with human skin and which received PBS instead of huPBMC showed no evidence of inflammation in either epidermis or dermis (**Fig. 1A**).

Inflammation of lymphocytes is regulated by pro-inflammatory cytokines and chemokines that attract these immune cells. To assess if human cytokines and chemokines might be involved in the development of skin inflammation in the huPBL-SCID/skin allograft model we determined gene expression levels in the skin grafts using quantitative real time PCR (qPCR). To this end the human skin grafts were removed 21 days after infusion of PBMC, at this time point clear inflammation hallmarks were observed by histology and immunohistochemistry (**Fig. 1** **,** **2A**). From the centre of the human skin grafts 4 mm punch biopsies were taken and subsequently prepared for qPCR analysis. A clear increase in gene expression of the proinflammatory human cytokines IL1B, IL6 and IL8 was observed in the PBMC-injected skin compared to the PBS-treated controls (**Fig. 2B**). Also, we found increased gene expression of the human chemokines CXCL1, CXCL10 and CCL5, as well as DEFB4, the gene encoding the antimicrobial peptide human beta defensin-2 (hBD2) which was recently demonstrated to attract CCR6 expressing cells [16]. mRNA levels of human cytokines exclusively or predominantly expressed by immune cells were close to or below the detection limit in either some of the PBS-injected controls (e.g. IFNγ which is clearly induced in the treated skin) or in both treated and control skin samples (such as IL12B, IL23A, IL17A, IL22 and IL20)

### Infiltration of CD4+ and CD8+ IL-17A-producing T cells and CD4+ Foxp3-expressing T cells in the inflamed human skin

**Figure 3:**
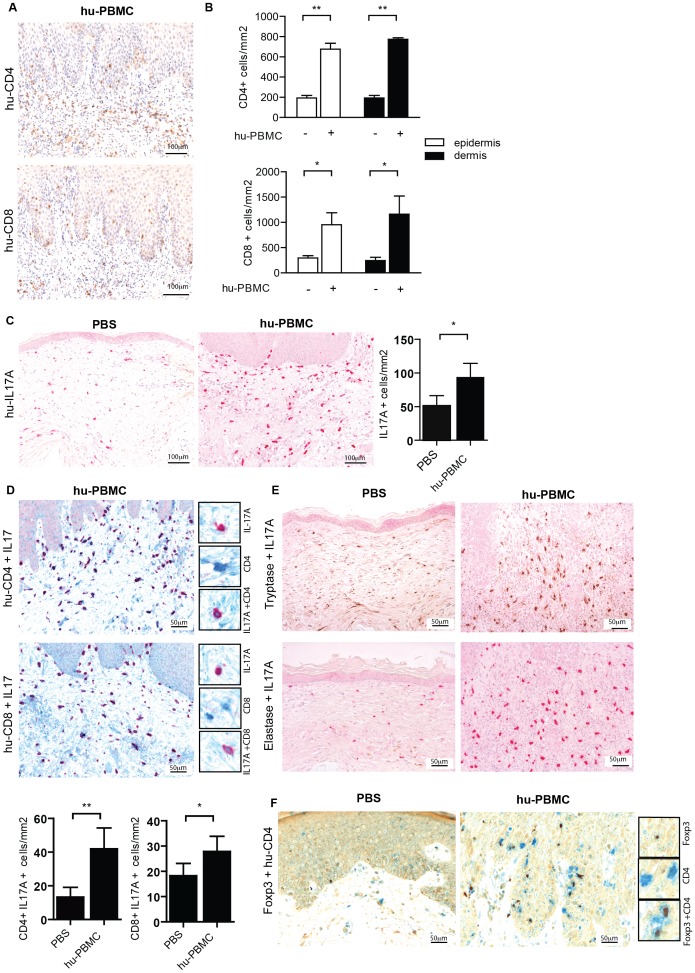
Infiltration of human IL-17A-producing T cells and CD4+ Foxp3-expressing T cells in the inflamed human skin in the SCID/skin allograft mouse model. **A.** Immunohistochemistry of human CD4 (brown, top) and CD8 (brown, bottom) expression in human skin grafts from SCID beige mice 21 days after infusion or huPBMC. Photographs show representative examples. 20× magnification. **B.** Summarized data of figure A. showing mean±SEM CD4 (top) or CD8 (bottom) positive cells/mm^2^ of n = 4 and 10 upon PBS and huPBMC infusion resp in the epidermis (white bars) and dermis (black bars). **C.** Immunohistochemistry of human IL-17A expression in human skin grafts from SCID beige mice 21 days after infusion of PBS (left) or huPBMC (right). Photographs show representative examples of n = 6 (huPBMCs) n = 3 (controls). 20× magnification. Graph shows summarized data of IL-17A positive cells/mm^2^ following PBS or huPBMC infusion in the human skin biopsies (mean±SEM, of n = 4 and 10). **D.** Immunohistochemistry of coexpression of human CD4 (blue) and IL-17A (red, top) and CD8 (blue) and IL-17A (red, bottom) in human skin grafts from SCID beige mice 21 days after infusion of huPBMC (20× magnification). Inserts show a higher magnification (40×) of single CD4/CD8 and IL-17A staining and CD4/IL-17A or CD8/IL-17A co-staining. . Photographs show representative examples of n = 6. **E.** Immunohistochemistry of IL-17A (red) in human mastcell tryptase (brown) and granulocyte elastase (brown) in human skin biopsies from SCID beige mice 21 days after infusion of PBS (left) or huPBMC (right). Photographs show representative examples of n = 6 (huPBMCs) n = 3 (controls). 20× magnification. **F.** Immunohistochemistry of co-expression of human Foxp3 (brown) and CD4 (blue) in human skin grafts from SCID beige mice 21 days after infusion of PBS (left) or huPBMC (right) (40× magnification). Inserts show a higher magnification (63×) of single Foxp3 and CD4+ staining and Foxp3/CD4 co-staining. Photographs show representative examples of n = 6 (huPBMCs) n = 3 (controls).

Next, we further characterized the mononuclear cell infiltrate in the skin by immunohistochemistry, and focused our analysis on quantification of human CD4+ and CD8+ T cells. As reported previously on the huPBL-SCID/skin allograft model [5], [6] the human skin contained both CD4+ and CD8+ T cells (**Fig. 3A**). CD4+ T cell infiltrates were predominantly present within the dermis (296,56±86,52 vs 954,68±35,9 cells/mm^2^, PBS vs huPBMC, respectively, p<0,01), while CD8+ T cells were present in both dermis (245,42±10,3 vs 1162,85±107,8 cells/mm^2^ p<0,05) and epidermis (296,6±86,5 vs 954,7±309,4 cells/mm^2^ p<0,05) (**Fig. 3B**).

The cytokine interleukin 17A, which amongst other cell types is produced by T cells, has strong pro-inflammatory potential and has been clearly associated with psoriasis [17]–[19], and it also contributes to the pathology of atopic dermatitis [20], [21]. Therefore, we analyzed the *in vivo* potential of the human CD4+ and CD8+ T cells to produce IL-17A in this huPBL/SCID model. IL-17A-producing cells were clearly present in the dermis, at the basal layer and also in the epidermis (**Fig. 3C**); both human CD4+/IL-17A+ and CD8+/IL-17A+ (41.95±12.38 and 27.8±5.97 cells/mm2, resp.) were observed (**Fig. 3D**). Next to these IL-17 –producing T cells, also CD3 negative IL-17-producing cells were observed. Mast cells and neutrophils are known for their IL-17A producing potential [22]. Here, we could clearly demonstrate that in the inflamed human skin human IL-17 production was associated with tryptase+ human mast cells, while we could not demonstrate IL-17+ elastase+ human neutrophils (**Fig. 3E**). We could not demonstrate IL-17A in the serum of the mice 3 weeks after infusion of huPBMC (data not shown), which suggests that IL-17 plays a predominant role in the local inflammatory skin response.

As opposed to pro-inflammatory cells, anti-inflammatory cells are needed to regulate the immune response. CD4+ regulatory T cells that express the transcription factor Foxp3 are renowned for their immunosuppressive potential and control of proinflammatory T cells. Therefore we analyzed the presence of human Foxp3+ cells in the human skin of the huPBL-SCID-huSkin model. CD4+ Foxp3+ cells were mainly present at the basal layer (**Fig. 3F**).

Taken together, inflammation of the human skin transplant of the huPBL-SCID-huSkin model is characterized by aberrant epidermal differentiation, presence of human cytokines and chemokines and influx of CD4+ and CD8+ T cells and mast cells that are able to produce the proinflammatory cytokine IL-17 as well as the presence of putative anti-inflammatory Foxp3 expressing CD4+ T cells.

### Local human skin inflammation in the huPBL-SCID-huSkin model is paralleled by systemic CD4 and CD8 T cell activation, proliferation and acquisition of homing markers

In addition to the analysis of the local inflammatory response in the human skin, we studied the systemic response of the CD4+ and CD8+ T cells in the huPBL-SCID-huSkin model. Mice were transplanted with human skin that was allowed to heal for 3 weeks and subsequently inoculated with 150×10^6^ human PBMC (i.p.) as describe above. After 3 weeks the CD4+ and CD8+ T cells were enumerated in peripheral blood, spleen and lymph nodes, and these cells were analyzed for cell division status, activation and homing marker expression using flowcytometry.

**Figure 4:**
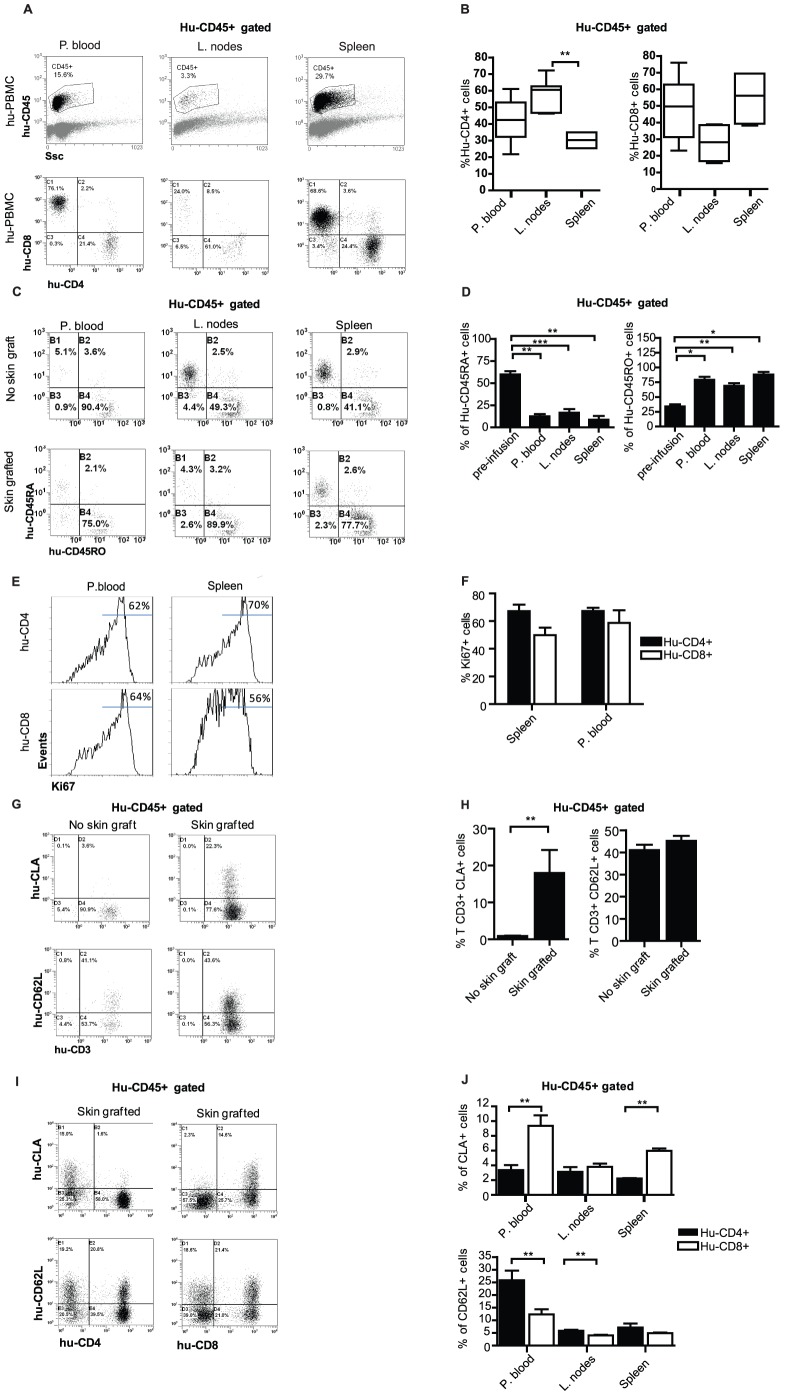
Systemic human CD4 and CD8 T cell activation, proliferation and acquisition of homing markers in the SCID/skin allograft mouse model. **A.** Flowcytometry showing side scatter (SSC, X-axis) and CD45 expression (Y-axis) (top panel) and CD4 (X-axis) and CD8 (Y-axis) expression (lower panel) in peripheral blood and lymph node and spleen cell suspensions 21 days after infusion or huPBMC. Representative dotplots are shown (n = 4–7). CD4/CD8 expression is shown after gating on huCD45+ cells. In case of PBS infusion hardly any human CD45+ lymphocytes was detected (data not shown). **B.** Summarized flowcytometry data of human CD4+ and CD8+ cells upon huPBMC infusion as shown in A. (n = 6). **C.** Flowcytometry of human CD45RO+ (X-axis) and CD45RA+ (Y-axis) cell populations in peripheral blood and lymph node and spleen cell suspensions 21 days after infusion of huPBMC in SCID beige mice that either lacked (top panel) or were previously grafted with human skin (lower panel) Representative dotplots gated on huCD45+ cells are shown. **D.** Summarized flowcytometry data of CD45RO+ and CD45RA+ cells upon huPBMC infusion in SCID beige mice that were grafted with human skin as shown in C. (n = 6). **E.** Flowcytometry of Ki67 expression in CD4+ (top panel) and CD8+ (lower panel) human T cells in peripheral blood (left) and spleen (right). **F.** Summarized flowcytometry data of Ki67 expression in CD4+ and CD8+ human T cells as shown in E. (n = 6). **G.** Flowcytometry of CLA (top panel) and CD62L (lower panel) expression on human CD3+ cells populations in peripheral blood, 21 days after infusion of huPBMC in SCID beige mice that either lacked (right panel) or were previously grafted with human skin (right panel) Representative dotplots gated on huCD45+ cells are shown. **H.** Summarized flowcytometry data of CLA and CD62L expression on human CD3+ cells as shown in G. (n = 4–5). **I.** Flowcytometry of CLA (top panel) and CD62L (lower panel) expression on human CD4+ (left) and CD8+ (right) cells in peripheral blood, 21 days after infusion of huPBMC in SCID beige mice that previously grafted with human skin. Representative dotplots gated on huCD45+ cells are shown. **J.** Summarized flowcytometry data of CLA and CD62L expression on human CD4+ and CD8+ cells as shown in peripheral blood, lymph nodes and spleen. (n = 4–5).

Anti-human CD45 monoclonal antibody was used to detect human lymphocytes in the humanized mice. CD45-expressing CD4+ and CD8+ T cells were observed in the peripheral blood, spleen and lymph nodes of the huPBL-SCID-huSkin model (**Fig. 4A**). In the peripheral blood equal percentages of CD4+ (42,3±5,5%) and CD8+ (48,5±7,6%) T cells were found (**Fig. 4A**), whereas in the lymph nodes CD4+ T cells (58,9±3,7%) were more predominant as compared to CD8+ T cells (27,7±3,9%)(**Fig. 4B**). Although less clear, the spleen seemed to contain more CD8+ (55,0±6,9%) than CD4+ T cells (30,2±2,0%) (**Fig. 4B**).

The majority of human T cells present in spleen, lymph nodes and peripheral blood expressed the memory marker CD45RO (**Fig. 4C,D**). Given that the inoculated huPBMC population pre-infusion contained about 60% CD45RA expressing T cells (**Fig. 4D**), which is indicative for the presence of naïve T cells, this suggests that the naïve T cells became activated *in vivo*. The presence of an allogeneic human skin transplant was required to induce this activated phenotype *in vivo*, as in the absence of a human skin transplant higher number of naïve CD45RA+ were observed spleen and lymph nodes (**Fig. 4C**). Irrespective of whether human skin was grafted, in peripheral blood we found similar percentages (>90%) of CD45RO+ cells (**Fig. 4C**). It should however be emphasized that higher absolute CD45RO+ numbers were found when a skin transplant was present (approx. 5×10^4^ vs. 1×10^2^ CD45RO+ cells in 20 µl *retro-orbital blood samples* after human skin transplantation vs. no transplantation, resp.) In peripheral blood, and spleen we found proliferating human CD4+ and CD8+ cells, as indicated by Ki67 staining (**Fig. 4E,F**). Interestingly, we observed that the presence of a human skin graft led to the induction of cutaneous lymphocyte associated antigen (CLA) expression on peripheral CD3+ T cells (**Fig. 4G,H**). This was not the case for the lymph node homing marker CD62L (**Fig. 4G,H**). It appeared that in particular CD8+ T cells had acquired CLA expression which was observed in both peripheral blood cells and spleen, but not in the case of lymph nodes (**Fig. 4I,J**). This indicates that the human skin can instruct homing receptor expression of the inoculated human cells in this humanized mouse model. Moreover, we found that more CD4+ T cells as compared to CD8+ T cells in the peripheral blood expressed the lymph node homing marker CD62L (**Fig. 4I,J**).

In summary, the flowcytometric analysis of human CD4+ and CD8+ T cells in peripheral blood and lymphoid organs in the huPBL-SCID-huSkin humanized mouse enables the study of the immune status and homing of human T cells *in vivo*. This together with the study of the local inflammatory response, as described above, empowers the huPBL-SCID-huSkin model as an important tool in the development and preclinical evaluation of novel systemic immunomodulating agents.

### Cyclosporin-A and rapamycin prevent human T cell mediated skin inflammation and systemic activation and proliferation of the human T cells

Next, we studied the value of the above mentioned newly identified parameters in the huPBL-SCID-huSkin allograft model by analyzing the effect of systemic treatment with the T cell inhibitory agents cyclosporine-A and rapamycin. We used the combination of Cyclosporine (CsA) and Rapamycin (Rapa), which has previously been demonstrated to decrease the extent of mononuclear cell infiltration and reduce the degree of microvessel injury in a huPBL-SCID-huSkin model [6]. Administration of CsA (0,4 mg/kg/day) was started at the day of inoculation with the huPBMC. Co-administration of Rapa (0,4 mg/kg/day) was initiated at day 7 and given on alternate days until the end of the immunosuppressive treatment.

**Figure 5:**
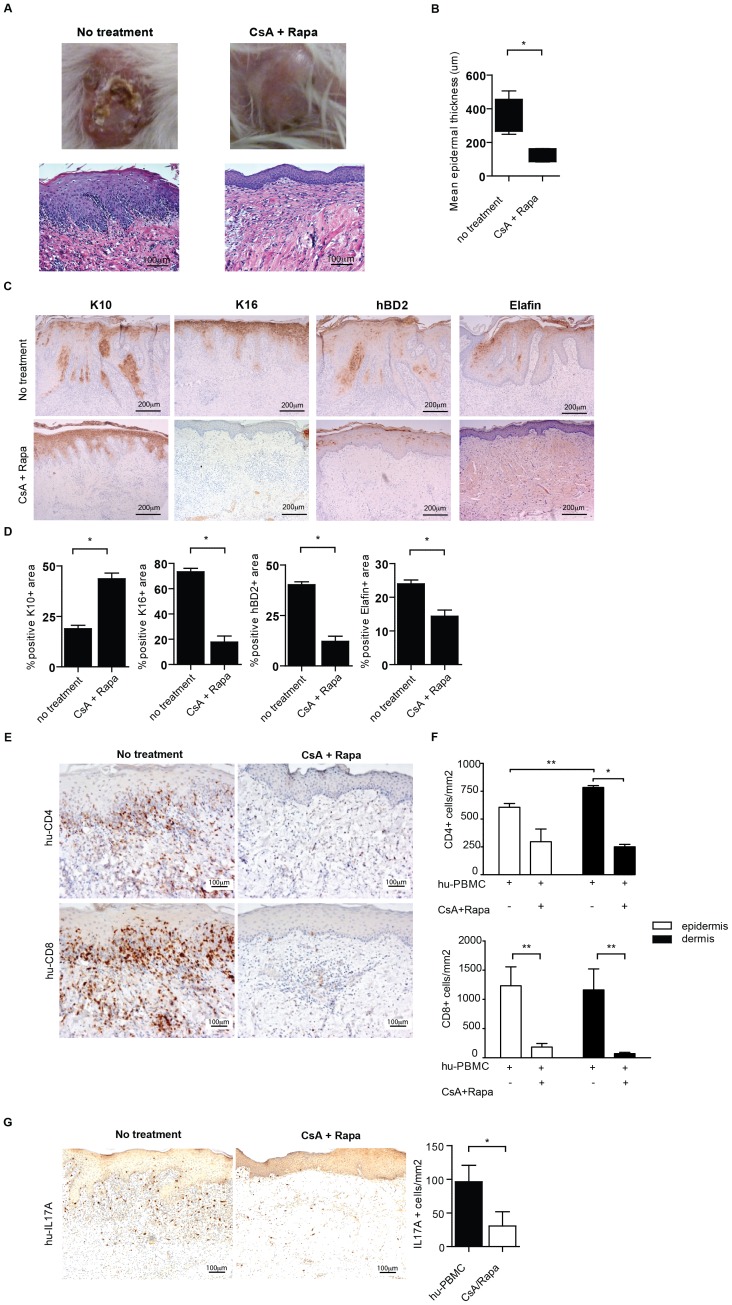
Cyclosporin-A and rapamycin treatment restores aberrant human epidermal differentiation marker expression and prevents infiltration of inflammation associated IL-17A producing human T cells of the human skin in the huPBL-SCID/skin allograft mouse model. **A.** Macroscopic (top panel) and histological (H&E) (lower panel) appearance of human skin graft from SCID beige mice 21 days after infusion of huPBMC with no treatment (left panel) and treatment with CsA and Rapa (right panel). **B.** Quantitative microscopic histological analysis of the epidermal thickness (µm) of skin grafts following infusion of huPBMC with or without CsA and Rapa. Mean±SEM are shown for n = 3 and 5 upon PBS and huPBMC infusion resp. 20× magnification. **C.** Immunohistochemistry of K10, K16, hBD2 and Elafin in human skin grafts from SCID beige mice 21 days after infusion (i.p.) of huPBMC without (top panel) and with CsA and Rapa treatment (lower panel). Representative examples are show. 10× magnification. **D.** Summarized data represented in C are given in the figures. Mean±SEM percentages of the area positive for the indicated markers is shown for n = 3 and 4–6 upon no treatment and treatment with CsA and Rapa, resp. **E.** Immunohistochemistry of CD4 (top panel) and CD8 (lower panel) in human skin grafts from SCID beige mice 21 days after infusion (i.p.) of huPBMC without (left panel) and with CsA and Rapa treatment (right panel). Representative examples are show. 20× magnification. **F.** Summarized data represented in E. are given in the figures. Mean±SEM percentages of CD4 (top) and CD8 (bottom) positive cells is shown for n = 3 and 4–5 upon no treatment and treatment with CsA and Rapa, resp. **G.** Immunohistochemistry of IL-17A (brown) (representative examples of n = 5 are shown) 10× magnification. Graph shows summarized data of IL-17A positive cells/mm^2^ present in human skin biopsies following infusion of huPBMC in the absence or presence of Rap/CsA resp. (mean±SEM, of n = 3 and 4–5).

Macroscopic evaluation 21 days after inoculation with huPBMC indicated that treatment with CsA and Rapa reduce visible signs of inflammation; the skin graft looked healthier and less inflamed, as indicated by a reduction in erythema, scaling and skin thickness (**Fig. 5A**). Histological analysis revealed that the epidermal thickening, elongated rete ridge formation and mononuclear cell infiltrates induced in this humanized mouse model was effectively inhibited by combined CsA and Rapa treatment (**Fig. 5B**). Quantitative microscopic analysis clearly demonstrated a significant reduction in epidermal thickening by CsA and Rap treatment (315,9±68,2 vs 135,4±45,1 µm, no treatment vs CsA+Rapa, resp, p<0,05)(**Fig. 5B**). Immunohistochemistry revealed that the aberrant epidermal differentiation, as indicated by the increased hBD2 and Elafin expression, and dysregulated K10/K16 expression, in the human skin were significantly inhibited following CsA and Rapa treatment (hBD2; 40,2±2,9 vs 12,2±5,2, Elafin; 24±2 vs 14,4±4,K10; 18,9±3,4 vs 43,6±6,8, K16; 73,3±4,7 vs 17,8±10% positive area, no treatment vs CsA+Rapa, resp, p<0,05) (**Fig. 5C,D**). As expected, the number of CD4+ and CD8+ T cells in the human dermis (CD4; 783,5±38,8 vs 251,1±42,9, CD8; 1162,9±716,3 vs 71,3±38,4 cells/mm^2^, no treatment vs CsA+Rapa, resp, p<0,05 and p<0,01) and epidermis (CD4; 605,8±76,6 vs 296,6±35,6, CD8; 1232,5±648,4 vs 183,9,9±10,7 cells/mm^2^, both p<0,01) were significantly reduced by the immunosuppressive treatment (**Fig. 5E,F**). Interestingly, the numbers of IL-17A-producing T cells were also substantially inhibited (**Fig. 5G**). CsA+Rapa treatment resulted in a substantial reduction of Foxp3+ cells in both the human skin graft and spleen (data not shown).

**Figure 6:**
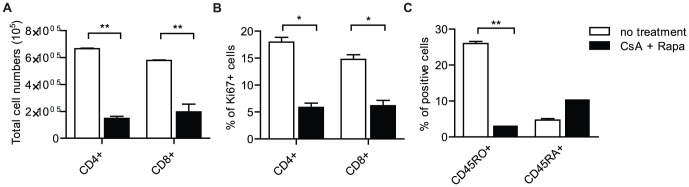
Cyclosporin-A and rapamycin treatment prevents systemic activation and proliferation of human CD4+ and CD8+ T cells in the huPBL-SCID/skin allograft mouse model. **A.** Enumeration of CD4+ and CD8+ cell populations by flowcytometry in peripheral blood 21 days after infusion of huPBMC without (white bars) or with CsA (black bars) and Rapa. Summarized flowcytometry data are presented (n = 6). **B.** Flowcytometry of Ki67 in human CD4+ of CD8+ cells in peripheral blood 21 days after infusion of huPBMC without (white bars) or with CsA and Rapa (black bars). Summarized data of n = 6 are shown. **C.** Flowcytometry of CD45RO and CD45RA expression on human CD3+ T cells in peripheral blood after infusion of huPBMC without (white bars) or with CsA and Rapa (black bars). Summarized data of n = 6 are shown.

Next, using flowcytometry, we analyzed the effect of CsA and Rapa treatment on the systemic immune response. We found that following CsA and Rapa treatment the number of human CD4+ and CD8+ T cells was reduced in peripheral blood of the mice (CD4+, 6,66×10^5^±6,08×10^3^ vs 1,47×10^5^±2,78×10^4^ and CD8+, 5,78×10^5^±5,51×10^3^ vs 1,96×10^5^±1,00×10^5^ total cell numbers, no treatment vs CsA+Rapa, resp. (p<0,01) **Fig. 6A**).

Moreover, after CsA and Rapa treatment the number of proliferating (Ki67 expressing) (**Fig. 6B**) and activated/differentiated (CD45RO expressing) (**Fig. 6C**) human CD4+ and CD8+ T cells in the peripheral blood was substantially decreased.

## Discussion

In our current work we further advanced the huPBL-SCID-huSkin allograft model initially described Pober's group [5], [6] for studying local skin inflammation, by identifying new local (skin) – as well as systemic parameters that turn this model into an even more powerful tool to study human skin immunopathology *in vivo*. A good understanding of the human skin immunopathology is crucial in the development of novel therapeutics for the treatment of (auto) inflammatory skin diseases.

Although the major focus of the huPBL-SCID-huSkin allograft model is on skin immune pathology, we also directed our attention on the systemic immune response of the human PBMC. We here demonstrate systemic proliferation, activation, and induction of homing marker acquisition by human T cells. This was prevented by systemic treatment with the immunosuppressive agents CsA and Rapa. Systemic analysis of the immune response in this preclinical humanized model is important for two reasons. First, the induction of a local T cell immune response is a multistep phenomenon taking place locally at the affected skin site (antigen recognition), in the draining lymph nodes (antigen presentation, T cell activation, differentiation, expansion) and peripheral blood (migration of activated T cells to the affected site and to other lymph nodes) [14]. Second, during the last decade there is an increasing use of systemic agents, like anti-inflammatory drugs or biologicals, to treat severe inflammatory skin diseases [23] [24]. At present, little Information is available on the effects of systemic medication of immune modulating agents and their effects on the systemic immune response in humanized mouse models. This information is crucial to identify treatment-related systemic biomarkers for immunomonitoring of patients undergoing clinical trials. For analysis of the systemic human immune response in the huPBL-SCID-huSkin model, peripheral blood samples containing relatively few cells can be used to analyze the effects of an anti-inflammatory drug by multi-color flowcytometry over time.

In the huPBL-SCID-huSkin model we observed that the presence of the human skin graft instructs CLA expression on a fraction of the peripheral human CD8+ T cells. This suggests that migration of T cells from skin or skin draining lymph nodes to the periphery is taking place. In mice it has been demonstrated that memory/effector phenotype T cells migrated from the skin to the draining lymph nodes in the steady state conditions, and this process was increased during a cutaneous immune response [25]. Early in a primary cutaneous immune response, proliferating T cells were released from the skin-draining lymph nodes and migrated via the circulation to antigen free lymph nodes that drain other tissues [26]. This migration of dividing T cells from the skin draining lymph nodes might explain the presence of human dividing Ki67+ T cells that we identify in the peripheral blood and spleen in the huPBL-SCID-huSkin model. Little is known about the migratory capacity of human T cells *in vivo*, the huPBL-SCID-huSkin model will be a helpful tool in improving our understanding in this respect.

IL-17-producing CD4+ T cells, designated Th17 cells, are important in the protection against extracellular pathogens, but Th17 cells are also associated with inflammatory and autoimmune conditions in humans [27]. IL-17 is a cytokine that acts as a potent proinflammatory mediator by increasing chemokine production to recruit monocytes and neutrophils to the site of inflammation. Immunity mediated by Th17 cells is particularly important at epithelial and mucosal surfaces [28]. IL-17-producing T cells appear to be important in the pathogenesis of psoriasis; Th17 cells have been demonstrated by both immunohistochemistry and flowcytometry in psoriatic lesions [17], [18]. Furthermore, a recent phase-II clinical study with a human antibody to IL-17A (AIN457, secukinumab) in psoriasis patients showed promising results supporting a role for Th17 in the pathophysiology of psoriasis [19]. Also in the early stages of atopic dermatitis IL-17-producing cells seem to contribute to the pathology of the disease [20], [21]. In the inflamed skin of the huPBL-SCID-huSkin model we observed the presence of IL-17A-producing CD4+ and CD8+ cells. Although Th17 cells, which are classically defined as CD4+ IL-17-producing cells, previously has received the major focus, it has recently been established that also CD8+ IL-17-producing cells contribute to inflammatory skin disorders including psoriasis [29], [30].

Regulatory T cells are important in the control of immune homeostasis and tolerance [31]. In particular CD4+ regulatory T cells expressing the Treg master transcription factor Foxp3 were extensively studied over the last decade. Skin resident CD4+ Foxp3+ Treg were identified in mice and men [25], [32]. In mice, skin resident CD4+Foxp3+ Treg suppress inflammation and appear to migrate from the skin to the draining lymph nodes under steady state conditions. During a cutaneous immune response migration of Treg was increased [25], [32]. Moreover, Treg that migrated from the skin returned to the skin upon skin antigen exposure [25]. In normal human skin under steady state conditions between 5 and 10% of the skin resident T cells are CD3+/FOXP3+ Treg [33] and proliferation of CD4+/Foxp3+ Treg was induced after a cutaneous challenge [34]. Together these data suggest that Treg circulate between blood, skin, and lymphoid tissues and that local Treg proliferation takes place in order to regulate peripheral skin immune responses. In the affected human skin of the huPBL-SCID-huSkin model, we now demonstrate the presence of human CD4+Foxp3+ T cells in the dermis of the human skin, which suggests active immune regulation in the inflamed human skin of this humanized mouse model. A possible reason why Foxp3+ Treg in this model do no inhibit the IL-17A production by CD4+ and CD8+ T cells might be explained by the fact that Treg can become unstable under proinflammatory conditions and lose suppressor function and even gain pro-inflammatory characteristics, resulting in an increased inflammatory response [35]. Also human Treg have this propensity to convert into proinflammatory cytokine secreting cells, in particular when activated under proinflammatory conditions [36].

We studied gene expression levels of cytokines and chemokines by quantitative real-time PCR in the affected human skin of the mice 3 weeks after the inoculation with PBMC when inflammation of the human skin was established. We found an increase in gene expression of human IL1B, IL6, IL8, CXCL1, CXCL10, CCL5 and DEFB4. The upregulation of DEFB4 correlated with the increased protein levels of hBD2 found by immunohistochemistry. Besides the chemokines and cytokines presented in figure 2C, we analyzed a number of other relevant cytokines that might play a role in skin inflammation (such as IL12, IL23, IL17, and IL22). Gene expression levels of these cytokines, arising from immune cells, were close to or below the detection limit. The reason for this is probably related to the fact that we have been analyzing gene expression at a late time point when overt inflammation was taken place (3 weeks after infusion of human PBMC) combined with the low levels of immune cells compared to keratinocytes in the skin biopsy. Future studies are needed to reveal time-kinetics of gene expression.

In the humanized inflammatory skin model that we here present, we showed differential expression of epidermal pathology-related proteins such as increased human β-defensin-2, Elafin, Keratin 16 (K16), and Ki67 levels, and reduced expression of Keratin 10 (K10). Most chronic inflammatory skin diseases are characterized by increased proliferation of keratinocytes, resulting in epidermal thickening (acanthosis). Proliferating keratinocytes are further characterized by upregulation of the proliferation marker Ki67. The proliferative response is further accompanied by a shift in keratin expression in the supra-epidermal layers. K16 is upregulated, while K10 is downregulated in keratinocytes. K16 expression, which is not expressed in healthy epidermis, was first explained as the specific result of hyperproliferation. However, more recent studies show K16 expression to be a marker of general trauma and stress of the skin ([37], [38]. Elafin and hBD-2 are anti-microbial peptide/proteins secreted by keratinocytes, they constitute part of the innate immune defense and participate in skin protection against invading microorganisms [39]. Elafin and hBD-2 are not present in healthy human epidermis, but they are highly induced under chronic inflammatory conditions of the skin (eg. psoriasis) and in case of skin barrier disruption [40]–[43]. The proinflammatory cytokine IL-17A can induces the expression of hBD2 in keratinocytes [44]–[46]. Next to its antimicrobial activity, hBD2 acts as pro-inflammatory chemoattractant for immune cells such as T-cells, dendritic cells, mast cells and neutrophils [47]. The upregulation of these anti-microbial peptides in our model is most likely the result of the influx of activated T cells and other immune cells and points to involvement of the innate immune system and impaired skin barrier function.

Psoriasis is a highly prevalent T cell mediated chronic inflammatory skin disease, which has both environmental and genetic causes to its etiology [48], [49]. The multi factorial and complex pathophysiology of the disease results in disturbed communication between cells of the immune system and epidermal cells, leading to abnormal differentiation and hyper- proliferation of keratinocytes [50]. Recently it emerged that the disease is strongly associated with IL-23 [51] and IL-17-producing T helper cells [17]–[19]. Psoriatic plaque lesions are histologically characterized by an increase in epidermal thickness, caused by disturbed keratinocyte differentiation and hyper proliferation (acanthosis), elongated epidermal rete ridges and influx of immune cells among which many T cells [50]. Clinically these alterations are represented by scaling, plaque thickening, and erythema [50]. At the molecular level, a regenerative epidermal differentiation program is induced that includes expression of genes such as Keratin 16 (K16), elafin, psoriasin and β-defensin-2 (hBD-2), and because differentiation is impaired keratinocytes loose expression of normal supra-basal keratin-10 (K10) [52]–[55] and increase expression of Ki67 in basal keratinocytes [50]. The association of psoriasis and hBD-2 induction [52] has recently been further substantiated by demonstrating increased β-defensin copy numbers in psoriasis patients [42].

In the inflamed human skin of the huPBL-SCID-huSkin model we here demonstrate that the skin inflammatory phenotype resembles human plaque-type psoriasis at multiple levels; macroscopically we found erythema and skin thickening, microscopically using histology we demonstrated acanthosis, parakeratosis and psoriasis like rete ridges, and by immunohistochemistry we found increased expression of hBD-2, elafin, K16 and Ki67 and reduced K10 expression. Moreover, we detected CLA expressing human CD8+ T cells in the peripheral blood of the huPBL-SCID-huSkin model. The presence of CLA-expressing CD8+ T cells in the peripheral blood of psoriasis patients is a hallmark of the disease [56], [57]. Most of the huPBMC-induced changes were significantly inhibited or completely prevented after CsA and Rapamycin treatment. Together these findings suggest that the huPBL-SCID-huSkin model is of potential interest to study the pathology of psoriasis at the level of skin and immune biology.

In conclusion, employing the huPBL-SCID-huSkin allograft model of human skin inflammation combined with the local and systemic markers for human cells that we here identified enable preclinical evaluation of novel immuno-modulating agents and cell-based therapy. Also, it will contribute to our understanding of inflammatory and regulatory processes by human T cells and keratinocytes in the pathology of skin inflammatory disorders *in vivo*.

## Materials and Methods

### Mice

Female B17.B6-*Prkdc^scid^Lyst ^bg^*/Crl (SCID/beige) mice, 6–8 weeks old, were purchased from Charles River Breeding Laboratories and housed in the SPF facility of the Central Animal Laboratory of the RUNMC. All the animal experimental procedures were in accordance with the international welfare guidelines taking in consideration the 3Rs (Refinement, Reduction, Replacement) and approved by the institutional ethical animal care committee of the Radboud University Nijmegen (number 2008167).

### Humanized mouse model; huPBL-SCID-hu Skin allograft model

The huPBL-SCID-hu Skin allograft model used in our study is with slight adaptations based on the model described by Murray et al. [5]. Superficial human skin, 600 to 700 µm thick, was harvested using a dermatome and kept in culture medium with penicillin/streptavidin at 4°C and within max. 30 hours transplanted onto the back of SCID/beige mice. Abdominal skin from healthy individuals was obtained from elective surgeries through the RUNMC Department of Plastic Surgery. After healing of the human skin (21 days), 150×10^6^ ficoll density gradient isolated (Lymphoprep; Nycomed-Pharma AS, Oslo, Norway) human peripheral blood mononuclear cells (huPBMC), obtained from buffy coats of blood donors, purchased from Sanquin Blood Bank, Nijmegen, The Netherlands, were infused intra peritoneally (i.p) in a volume of 0.8 ml PBS. Mice were killed at the end of the experiment and tissues of interest were collected.

The use of human skin and peripheral blood were approved and in accordance with the regulations set by the Medical Ethical Committees for human research of the RUNMC. Human skin and buffy coats from healthy donors, who gave written informed consent for scientific use of the human materials. Buffy coats were purchased from Sanquin Blood Bank, Nijmegen, The Netherlands

### Drug treatment

Cyclosporine–A (Sandimmune, Novartis®) was administered at a concentration of 0.4 mg/kg/day s.c. via mini-osmotic pump (pumping rate was 1 µl/h , reservoir volume 200 µl). The control group was implanted with a mini-osmotic pump filled with PBS. Rapamycin was injected intraperitoneally (i.p.) at a dose of 0.4 mg/kg body weight in a volume of 100 µL PBS at alternating days, starting 7 days after the infusion of huPBMC.

### Histology & Immunohistochemistry

Human skin grafts were fixed in neutral buffered 4% formalin (Mallinckrodt Baker, Inc Deventer, The Netherlands) for 4 hours, processed and embedded in paraffin. Sections (6 µm) were stained with hematoxilin-eosin (HE) or processed for immunohistochemical staining.

Keratinocyte differentiation was analyzed using primary, antibodies directed against: Elafin (rabbit 92-1), hBD-2 (ab9871, Peprotech, London,UK), K10 (RKSE60, Eurodiagnostica) and K16 (LL025, Novocastra Laboratories, Newcastle upon Tyne,UK). Cell division was studied using antibodies against Ki67 (MiB-1, Dako cytomation). To enumerate CD4+ and CD8+ T cells antibodies against CD4 (BC/F6, Santa Cruz Biotechnology, Santa Cruz, CA) and CD8 (144B, Dako Cytomation) were used. IL-17 production was detected using polyclonal goat IL-17A antibody (R&D-systems). To detect Foxp3 expression anti-FoxP3 (PCH101) was used. To detect the presence of human neutrophils and mast cells, antibodies against human neutrophil elastase (NP57; Dako) or human mast cell tryptase (AA1; Dako) were used. Antibody stainings were visualized using the Dako Cytomation EnVision+system-HRP (ABC) kit (DAKO, Glostrup, Copenhagen, Denmark) combined with 3,3′-diaminobenzidine tetrahydrochloride (DAB, brown) (Sigma-Aldrich, St. Louis, USA) or using that Labeled Streptavidin Biotin method (Universal LSAB Kit/AP; Dako) combined with either Permanent Red (Dako) or 5-Bromo-4-Chloro-3-Indolyl Phosphate/Nitro Blue Tetrazolium (BCIP/NBT) (Dako).

Sections were photographed using a microscope (Axiokop2 MOT; Zeiss, Sliedrecht, the Netherlands), digital camera (Axiocam MRc5; Zeiss) and AxioVision software (Zeiss).

### Image analysis of immunohistochemistry

To enumerate huCD4+ and huCD8+ cells, representative pictures were made at 10× magnification. A representative region of interest (ROI) was drawn from the lowest epidermal papilla till 300 µm dept into the dermis. Cell quantification was performed by setting a threshold and relating this to a number of cells per mm^2^. For evaluation of number of CD4+ and CD8+ IL-17A-secreting cells, double positively stained cells were counted manually in CD4 CD8 infiltrated areas of the tissues and the numbers were reported per mm^2^. Ki67+ cells were counted manually and related to the length of basement membrane (BM). For quantification of hBD-2, Elafin, K10 and K16 positive cells, photographs were made at 10× magnification. The total epidermal area and K10 or K16 positive area was measured in the ROI (epidermal compartment). Epidermal differentiation was defined as: % K10 or K16 positive epidermal area. Each photo was analyzed using ImageJ software (NIH, http://rsb.info.nih.gov/ij).

### Determination of epidermal thickness

Histologic assessment of the grafts was performed by light microscopy both before and after transplantation of human skin. The mean epidermal thickness was calculated using the program Visiopharm Integrator System (VIS) (Visiopharm, Hørsholm, Denmark) as epidermal area divided by epidermal surface length.

### Flowcytometry

Mesenteric/peripheral lymph nodes and spleen were processed to obtain a single cell suspension. Retro-orbital blood samples were collected and lymphocytes were isolated by density gradient centrifugation. For cell surface staining, the following conjugated mAbs were used: anti-CD45 PE (Beckman-Coulter), anti-CD3 PE (Beckman-Coulter), anti-CD4 PE-Cy7 (SFCI12T4D11, Beckman-Coulter), anti-CD8 PE-Cy5(B9.11 , Beckman-Coulter), anti-CD62L (DREG56, Beckman-Coulter) anti-CD45RA FITC (HI100, BD), anti-CD45RO ECD (UCHL1, Beckman-Coulter) and anti-CLA FITC (HECA-452, BD). Intracellular Foxp3 staining was performed after fixation and permeabilization of the cells as indicated by the supplier (Ebioscience). Cell samples were measured on a 5 color FC500 flowcytometer (Beckman-Coulter), and the data were analyzed using CXP software (Beckman-Coulter).

### Gene expression analysis

Tissues were homogenized using a TissueLyser (Qiagen). RNA was extracted using the RNeasy Lipid Tissue Mini Kit (Qiagen) and reverse-transcribed by use of the High-Capacity cDNA Reverse Transcription Kit (Applied Biosystems). The samples were amplified by quantitative real-time PCR using Applied Biosystems validated gene expression assays and PRISM7900HT sequence detection system (SDS 2.3). Expression of GAPDH was used for normalization and fold changes calculated by the comparative Ct method.

Gene expression assay ID: IL1B-Hs00174097_m1, IL6-Hs00985639_m1, IL8-Hs00174103_m1, CCL5-Hs00174575_m1, CCL20-Hs00171125_m1, CXCL1-Hs00236937_m1, CXCL10-Hs00171042_m1, DEFB4-Hs00823638_m1, IFNG-Hs00174143_m1, IL12B-Hs00233688_m1, IL23A-Hs00372324_m1, IL17A-Hs00174383_m1, IL22-Hs00220924_m1, IL20-Hs00218888_m1, GAPDH-Hs99999905_m1.

### Statistics

The results were analyzed by a two-tailed Mann Whitney t test using GraphPad Prism software. *, P value<0.05; **, P value<0.01; ***, P value<0.001.
